# Editorial: Advanced pre-clinical and pre-surgical assessment of musculo-skeletal medical devices

**DOI:** 10.3389/fbioe.2023.1264118

**Published:** 2023-07-21

**Authors:** Benedikt Helgason, Michael G. Bryant, Stephen J. Ferguson, Richard M. Hall

**Affiliations:** ^1^ Institute for Biomechanics, ETH Zurich, Zurich, Switzerland; ^2^ Future Health Technologies, Singapore-ETH Centre, Campus for Research Excellence and Technological Enterprise (CREATE), Singapore, Singapore; ^3^ School of Mechanical Engineering, Faculty of Engineering and Physical Sciences, University of Leeds, Leeds, United Kingdom

**Keywords:** medical devices, pre-clinical, pre-surgical, testing, *in silico*, *ex vivo*, *in vivo*

The orthopaedic implant market is worth in excess of €30B per annum globally with articulating joint replacements representing the largest orthopaedic sector. Growth in the sector of between 2% and 5% is expected over the near term due to the rapid demographic shift. Pre-clinical assessment, both experimental and *in silico*, is a series of necessary steps for the development, optimisation and validation of medical devices. It comprises the testing of implants using assessments that conform to agreed standards as well as those that are more bespoke and focus on the specific requirements and perceived usage of the device. However, once introduced the new technologies are not always successful clinically and may harm the patient. This may require an expensive intervention to correct the loss in the patient’s quality of life and greater mortality risk. These deficits in implant outcomes have brought into focus the role of pre-clinical simulation and the wider regulatory science that supports these activities.

The theme of the research included in this Research Topic, which is driven by our experience within the EU MSCA European Training Network, BioTrib, is how best to facilitate improvements in pre-clinical *in silico*, *in vitro* and *in vivo* testing within an orthopaedic context to allow a reduction in the adverse events that occur in implants once marketed. The studies cover techniques such as artificial intelligence, image processing, laboratory and computer simulations, to achieve this end.

Several of the studies are focused on addressing the limitations of using a “human in the loop” for, e.g., diagnosis or implant selection. Qu et al. used a deep learning approach for localizing cruciate ligament rupture on knee MRI images. The over all aim of such work is to identify the location and tissue quality of injured ACLs to determine if the ACL repair surgery can be performed. The goal of the work of Burge et al. was to develop a computational tool for automatic selection of total knee replacement implant size using X-ray images. Both studies support the use of algorithms to achieve repeatable outcomes to reduce operator dependency.

Some of the studies are comparing the biomechanical efficacy of two or more surgical procedures or devices, with the overall aim of ranking the procedures or improving device design. Here the benefit of preclinical *in silico* testing can be utilized to avoid subjecting patients to clinical trials of devices or procedures that may involve elevated risk. Fung et al., e.g., studied the efficacy of prophylactic ceramic-based cement augmentation of the proximal femur to prevent hip fracture. The authors provide insight into the potential efficacy of such a procedure, but prophylaxis for fracture prevention is a controversial Research Topic among clinicians. Using an advanced experimental model, Techens et al. studied the biomechanical consequences of cement discoplasty. This surgical procedure is relatively novel, and experimental models that can provide insight into, e.g., the changes in spine kinematics following the treatment, are needed.

Another important aspect of pre-clinical testing of orthopaedic devices is addressed in the study by Kohli et al. Here, a bioreactor analyses of tissue ingrowth, ongrowth and remodelling around implants is used to reduce the need for animal testing. Some of the studies in this Research Topic, fall under the category of novel measurement techniques and protocols. In the study of Kandel et al., an automated system is introduced for polymer wear debris analysis in total disc arthroplasty using convolution neural network. Using dual-plane fluoroscopy to observe treadmill walking, Zhou et al. investigate whether multi-planar instability, laxity and reduced knee flexion during the support phase of walking are determinants of return to sports. On the other hand, D’Isidoro et al. use dual plane moving fluoroscopy-based analysis, to study total hip arthroplasty kinematics during unrestricted activities of daily living. These advanced measurement techniques are providing novel insights into aspects of total joint replacements that are difficult to achieve with other, more conventional methods.

Overall, the papers on this Research Topic cover some recent advances in preclinical testing of musculoskeletal devices. The editors hope that this Research Topic will contribute to advancing the field of orthopaedics, by inspiring researchers, clinicians and industry alike, towards reduction in the need for animal testing, improving patient safety and lowering healthcare costs.

